# Mitigation of bio-corrosion characteristics of coronary artery stent by optimising *fs*-laser micromachining parameters

**DOI:** 10.1016/j.heliyon.2024.e28057

**Published:** 2024-03-15

**Authors:** Venkatesh Chenrayan, Dhanabal Palanisamy, Kalayarasan Mani, Kiran Shahapurkar, Manzoore Elahi M. Soudagar, Yasser Fouad, M.A. Kalam, Muhammad Mahmood Ali, Muhammad Nasir Bashir

**Affiliations:** a Polymer Composite Processing and Research Laboratory, Department of Mechanical Engineering, Alliance University, Anekal, Bengaluru, India; b Department of Mechanical Engineering, PSG College of Technology, Coimbatore, India; c School of Mechanical, Chemical and Material Engineering, Adama Science and Technology University, Adama, Ethiopia; d Institute of Power Engineering, Universiti Tenaga Nasional, Jalan IKRAM-UNITEN, 43000 Kajang, Selangor, Malaysia; e Division of Research and Development, Lovely Professional University, Phagwara, Punjab, 144411, India; f Faculty of Mechanical Engineering, Opole University of Technology, 45-758, Opole, Poland; g Department of Mechanical Engineering, Graphic Era (Deemed to be University), Dehradun, Uttarakhand 248002, India; h Department of Applied Mechanical Engineering, College of Applied Engineering, Muzahimiyah Branch, King Saud University, P.O. Box 800, Riyadh 11421, Saudi Arabia; i School of Civil and Environmental Engineering, FEIT, University of Technology Sydney, NSW 2007, Australia; j Centre for Mathematical Modelling and Intelligent Systems for Health and Environment (MISHE), Atlantic Technological University Sligo, Ash Lane, Sligo F91 YW50, Ireland; k Department of Mechatronic Engineering, Faculty of Engineering and Design, Atlantic Technological University, Sligo F91 YW50, Ireland; l Department of Mechanical Engineering, Yonsei University, 50-1 Yonsei-ro, Seodaemun-gu, Seoul, 03722, Republic of Korea; m Department of Mechanical Engineering, CEME, National University of Sciences and Technology NUST, Islamabad, Pakistan

**Keywords:** Nitinol, *Fs*-laser, Surface roughness, Volume ablation, Corrosion, GRA

## Abstract

Cardiovascular diseases, particularly coronary artery disease, pose big challenges to human life. Deployment of the stent is a preferable treatment for the above-mentioned disease. However, stents are usually made up of shape memory alloy called Nitinol. The poorer surface finish on the machined nitinol stents accelerates the migration of Nickel ions from the implanted nitinol stent, which is considered toxic and can lead to stenosis. The current study deals with controlling surface quality by minimising surface roughness and improving corrosion resistance. Femtosecond laser (*fs*-laser 10^−15^ s) micromachining was employed to machine the Nitinol surface to achieve sub-micron surface roughness. The Grey relational analysis (GRA)-coupled design of the experimental technique was implemented to determine optimal levels of four micromachining parameters (laser power, pulse frequency, scanning speed, and scanning pattern) varied at three levels to achieve minimum surface roughness and to maximise the volume ablation. The results show that to yield minimum surface roughness and maximum volume ablation, laser power and scanning speed are in a higher range. In contrast, the pulse frequency is lower, and the scanning pattern is in a zig-zag manner. ANOVA results manifest that scanning speed is the predominant factor in minimising surface roughness, followed by pulse frequency. Furthermore, the corrosion behaviour of the machined nitinol specimens was evaluated, and the results show that specimens with lower surface roughness had lower corrosion rates.

## 1 Introduction

Cardiovascular diseases pose a significant challenge to human life and are now the primary reason for medication and mortality worldwide [[1], [2], [3], [4], [5]]. Coronary artery disease (CAD) is the principal reason for death globally and poses a substantial financial burden and health anxiety on the human community [[6], [7], [8], [9], [10], [11]]. Because of plaque build-up under the endothelium, CAD is characterised by arterial constriction [[12], [13], [14], [15], [16]]. This criticality involves narrowing the artery lumen, which limits blood flow and results in inadequate delivery of oxygen and vitamins to the heart muscle. Stents are specialised medical devices that can be put into a narrowed channel under fluoroscopic or endoscopic guidance to maintain uniform blood flow and prevent potentially serious outcomes [12,17]. The initial generation of stents, known as BMS, were typically constructed from alloys such as stainless steel (316L), chromium-cobalt (Co–Cr), iridium-platinum (Pt–Ir), tantalum (Ta), and nitinol (Ni–Ti). Among these, stainless steel (316L) and nitinol are the most commonly employed materials [18,19]

Stent utilisation has become a preferred method for coronary artery treatment, as it helps reduce the danger of severe thrombosis and chronic restenosis. Traditional balloon-expandable stents, such as stainless steel stents, need to be pressurised dilatation to achieve optimal deployment in the atherosclerotic arterial walls. However, this can exacerbate endothelial denudation and vascular damage, increasing neointimal growth within the stents [20,21]. Furthermore, balloon-expandable stents have limitations, such as longitudinal shortening during expansion and limited flexibility. In contrast, self-expanding stents can take advantage of nitinol's super elastic properties, which enable them to achieve a sufficient expansion ratio, and reduced lengthwise shortening [1,7,11].

Nitinol is not only super elastic but also possesses corrosion resistance that is either equivalent to or better than stainless steel, and it is also biocompatible [6,10]. These qualities encourage the creation of such self-expanding devices, which can be used for stenting to treat artery disease (iliac, subclavian, renal, carotid and aortic intervention) [14]. Nitinol alloy is more biocompatible in the near term, but over time, a chance of migration of nickel ions from the substance which can be a threat to the immune system [2]. To overcome this, endothelial cells are needed on the stent surface [22], the surface with negligible roughness accelerates the growth of endothelial cells and retards the bio-corrosion [23]. Stents can be manufactured using both traditional and non-traditional machining processes. However, conventional machining could not be successful due to the submicron accuracy requirement of the stent and the inherent higher strength, hardness and chemical reactivity of the Nitinol [24]. Femtosecond laser micromachining is a highly suitable manufacturing method for nitinol stents due to its non-contact and low-heat nature, which enables the creation of intricate and precise geometries without causing any significant mechanical stress or excessive heat. This process characteristic makes it an excellent choice for preserving the unique properties of nitinol while manufacturing stents with intricate features and a high degree of accuracy [25]. Cheng-Shun Chen et al. [25] conducted a study on optimising laser processing for producing stents made of 316LVM stainless steel. The findings indicated a strong correlation between the surface roughness of the stent post-machining and the process parameters of laser machining.

A study by Raghavendra Rao et al. [26] explored the multi-response optimisation of Nd: YAG laser machining for thin superalloy sheets using GRA. The authors declared that the pulse frequency, pulse width, and cutting speed are significant parameters. K.K. Mandal et al. [27] experimented with the laser micro-machining of aluminium and its alloys, focusing on its application in aerospace, missile parts, and defence equipment. The study revealed that a rise in laser beam power leads to more surface roughness, while a rise in scanning speed results in lesser surface roughness. P. Deepu et al. [28] investigated femtosecond-based laser machining and the surface morphology of micro-holes in titanium alloy. The study revealed that the repetition rate, laser fluence, and pulse overlap ratio are the prime factors influencing surface roughness. Noorhafiza Muhammad et al. [29] conducted research on underwater and dry femtosecond laser processing of shape memory alloy tubes for the production of coronary stents. The study found that the surface imperfections and heat-affected zone were at a lower level in underwater than the dry environment.

### 1.1 Significance of the study

The increased usage of nitinol to fabricate the cardiovascular artery stent has become quite common. The dimensions and precision of the stent in the submicron range imply that micromachining is the right choice. Various literature studies reveal that the corrosion behaviour of the implanted stent poses a challenge to the bio-compatibility of the stent. However, the report concludes the surface roughness of the stent governs the corrosion resistance. This research work focuses on minimising the surface roughness and, in turn, the bio-corrosion behaviour of nitinol by optimising the micromachining parameters of *fs*-laser machining. The implementation of GRA coupled Taguchi mathematical model to arrive at optimal parameters to minimise the corrosion, the corrosion studies at a pH level equivalent to the human blood plasma, and the attempt in *fs*-laser machining of nitinol were declared to be the knowledge gap in this study. The prime novelty attributes about the study are as follows: 1. The machining of the cardiovascular stent in *fs*-laser machining and attainment of meagre surface roughness in the range of 10 nm. 2. The prediction of optimal parameters of *fs*-laser machining to achieve a minimal surface roughness (10 nm) consistently. 3. A clear establishment of a relation between surface roughness and biocompatibility.

## 2 Experimental details

### 2.1 Materials and processes

**Fig. 1:**
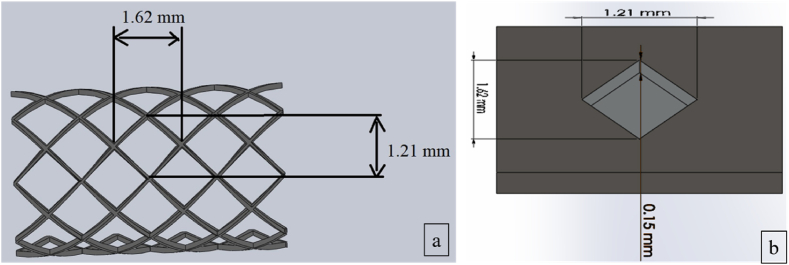
(a) Schematic diagram of the coronary stent, (b) Solid model of Micromachined single rhombus eye of the stent.

**Fig. 2:**
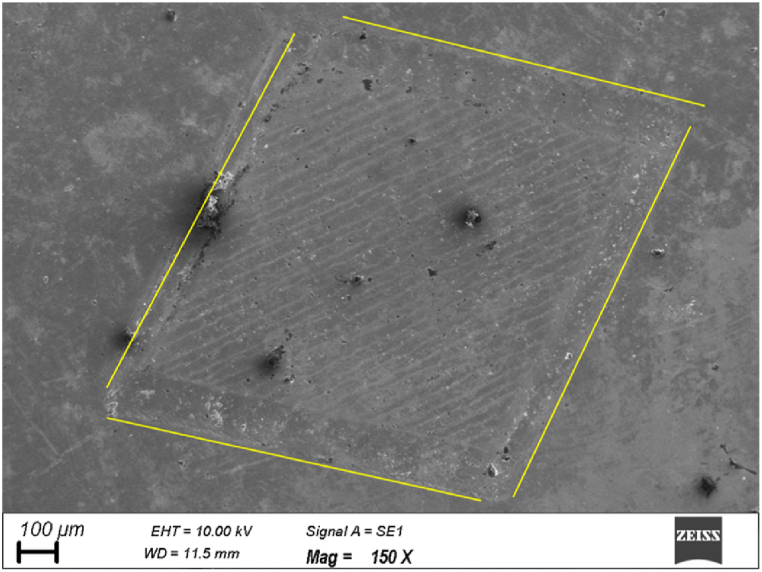
Micrograph of micromachined rhombus eye.

**Fig. 3:**
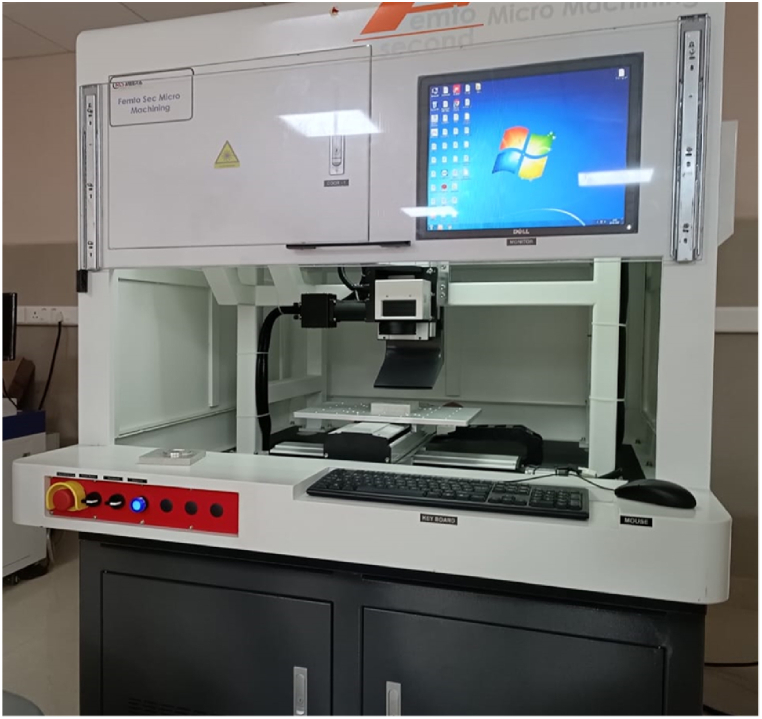
Femtosecond laser micromachining center

This work procured a nitinol plate of dimension 50 × 10 × 0.55 (all are in mm) from Nextgen steels and alloys. The chemical constituents and mechanical characteristics of nitinol are provided in Tables1 and 2, respectively. Fig. 1 (a) depicts the typical coronary stent machined using micromachining. Figs. 1 (b) and 2 depict the soild model and micrograph of the single rhombus eye of the stent micromachined from the nitinol plate using *fs*-laser micromachining respectively. Fig. 1 (b) highlights the blind depth of 0.15 mm micromachined in a stated diagonal dimension of 1.62 mm × 1.21 mm single rhombus. Three different laser scanning strategies, namely concentric, linear and zig-zag, were adopted to examine the volume ablation rate. Throughout the experiment, the laser beam of a diameter of 7 μm, hatch spacing of 0.03 mm and wavelength of 1035 nm were kept constant. This ultra-short pulse micromachining (Brand – Wuhan Huaray Precision Laser Co., Ltd, Model- HR-Femto-IR-50-40), as shown in Fig. 3, was employed to micromachining, which uses Titanium-Sapphire lasing medium, in which the laser energy distribution is Gaussian profiled. The detailed specification of the *fs*-laser micromachining resource is depicted in Table 3.

**Table 1:** Chemical composition of Nitinol.

Element	Nickel	Titanium
Composition (%)	50	50

**Table 2:** Mechanical characteristics of nitinol.

Ultimate strength (MPa)	Young's modulus (MPa)	Elongation at break (%)	Hardness (HRC)
1200	45–50	−20	60–65

**Table 3:** Specification of micromachining centre.

Max power	Max pulse width	Pulse frequency	Scanning speed	Focal length	Current
40 W	150 fs	1–800 kHz	1–1000 mm/s	170 mm	7 A

### 2.2 Surface roughness evaluation

Once machining trials were completed, the specimens were rinsed with acetone, and the topography was examined at eight different locations using the NTEGRA II Atomic Force Microscope. Two locations at the centre, four locations at the periphery and two locations between the periphery to the centre. The samples were first pasted to a metal bar using quick-drying cyanoacrylate glue. AFM probes with a curvature radius of 10 nm mounted on cantilevers of length 250 m and a spring stiffness of 0.1 N/m were employed for topographical characterisation. The obtained topographical images were subjected to surface roughness analysis using Gwyddion software, and the average surface roughness (Ra) data were used for further analysis.

### 2.3 Evaluation of volume ablation rate (metal removal rate)

The method of differential weighing enables simple determination of volume ablation rates by measuring the mass of material that has been ablated during laser machining [34]. This method involves measuring the weight loss of a material over a specific period while it is being exposed to the laser beam. The weight of each specimen ahead and later machining was weighed using standard analytical balance LX200ABL with a weighing capacity and accuracy of 120 g and ±0.003 g. Measured weight was used, along with laser machining parameters, to compute the weight loss of specimens after laser ablation. Volume ablation is computed using the following relation shown in (1), and Table 5 shows the computed values of the volume ablation rate.

(1) $${\;\tau }_{vol}=\frac{\;\Delta m\;V}{{\rho \;c\;k\;L}_{path}}$$

where, ${\;\tau }_{vol}$ is the volume ablation rate, Δm difference in weight before and after machining, V is scanning speed, ρ is the density of Nitinol, c is the pulse frequency, k is the number of passes, and ${\;L}_{path}$ length of the pass.

### 2.4 Evaluation of corrosion resistance

The corrosion behaviour of the nitinol specimens was conducted with the help of the electrochemical method, known to be the Tafel potentiodynamic polarisation technique. The corrosion behaviour of laser-ablated specimens was analysed in the Simulated Body Fluid (SBF) medium, which has a pH range closely resembling human blood plasma. The apparatus consists of an electrochemical cell assembled with the working, reference and a counter electrode connected to the potentiostat. A small incremental application of potential on the working electrode and the corresponding current was utilised to prepare the Tafel plot, which can help to determine the corrosion potential (E_corr_) and current density (I_corr_). Further, the corrosion rate was estimated with the following mathematical relation (2).

(2) $$Corrosion\;rate=\frac{0.129\;x\;I_{\text{corr}}}{Eq.wt\;x\;Density}$$

### 2.5 Strategy of experiments

The five process parameters of *fs*-laser micromachining were considered at three varied levels, as shown in Table 4. Since the surface roughness and volume ablation rate are the dependent properties of micromachining parameters, the level of parameters and their significance can be studied by implementing Taguchi's robust design [30–32]. Taguchi's L9 orthogonal array [33] is being implemented to conduct the machining trials. Table 5 depicts the experimental observation of both the objectives as per L9 orthogonal array experimental trials. Each trial was replicated thrice, and the average response value was used for further analysis.

**Table 4:** Process parameters and their levels.

Parameter	Unit	Level 1	Level 2	Level 3
Laser power	W	32	36	40
Scanning speed	mm/s	250	500	750
Pulse frequency	kHz	3	9	15
Scanning pattern	–	Concentric	Linear	Zig-zag

**Table 5:** Experimental trials and their response values.

Trial No	Control factors				Average response value	
	Laser power (W)	Scanning speed (mm/s)	Pulse frequency (kHz)	Scanning pattern	Surface roughness (nm)	Volume ablation rate (mm^3^/pulse)
1	32	250	3	Concentric	28.77	0.01004
2	32	500	9	Linear	19.73	0.00793
3	32	750	15	Zig-zag	19.117	0.00631
4	36	250	9	Zig-zag	20.158	0.0201
5	36	500	15	Concentric	16.99	0.0114
6	36	750	3	Linear	26.502	0.0021
7	40	250	15	Linear	13.171	0.0260
8	40	500	3	Zig-zag	27.62	0.0027
9	40	750	9	Concentric	18.33	0.0038

## 3 Statistical computation

### 3.1 Grey relational analysis (Gra)

Prof. Deng (1989) suggested that GRA can assist in resolving complicated issues despite the limited amount of accessible data [30]. The fundamental idea of GRA theory is the normalisation strategy. In addition, an exceptional feature of GRA is its capacity to address multi-response situations by transforming them into a single response [[31], [32], [33]]. To control the variability in the *fs*-laser micromachining process, it is necessary to reduce the impact of uncontrolled variables, also known as noise factors. This can be achieved analytically by utilising the signal-to-noise ratio (S/N), where the expected outcome of the responses, such as larger or smaller, plays a crucial role in determining the S/N ratio [34,35]. The present investigation focuses on quality characteristics, where the surface roughness should be minimum, and the volume ablation rate should be maximum. However, the present study limits with minimum trials (nine experiments) subject to resource availability. The minimum number of trials yield the data with uncertainties and variations. Presently, GRA is the only mathematical model to yield optimal solutions by handling unprecise data drawn from a smaller number of trials. Furthermore, the objectives involved in the study are distinct units. The normalisation step available in the GRA procedure merges the distinct units into a single Grey Relational coefficient (GRC), which is used to assess the performance weightage of each parameter. Hence, most of the other conventional or unconventional models limit the present problem not to be solved accurately. This logical hindrance restricts the study to limit with GRA. The basic steps involved in GRA include:

1) **Determining the input and output factors:** The primary parameters for the analysis are laser power, pulse frequency, scanning speed, and scanning pattern. The response factors are surface roughness and volume ablation rate.
2) **Computation of Signal-to-Noise (**S/N**) ratios:** The S/N ratios were computed for every trial of the experiment using equations (3), (4), which were chosen depending on the specific quality characteristic being analysed. For the smaller the better, case (3) is employed, and (4) is used for the larger the better case.

(3) $$\frac{S}{N}\;ratio=-10\;\log\frac{1}{n}\;\sum_{i=1}^{n}Y_{i}^{2}$$

(4) $$\frac{S}{N}\;ratio=-10\;\log\frac{1}{n}\;\sum_{i=1}^{n}\frac{1}{Y_{i}^{2}}$$

3) **Normalisation of data:** The input and output factors are usually measured in different units, so they need to be normalised to bring them to a common scale. This is done using relations (5) and (6) that convert the data to a scale of 0–1.

(5) $$Z_{ij}=\frac{Y_{ij}-\min\left(Y_{ij},i=1,2,\ldots ,n\right)}{\max\left(Y_{ij},i=1,2,\ldots ,n\right)-\min\left(Y_{ij},i=1,2,\ldots ,n\right)}$$

(6) $$Z_{ij}=\frac{\max\left(Y_{ij},i=1,2,\ldots ,n\right)-Y_{ij}}{\max\left(Y_{ij},i=1,2,\ldots ,n\right)-\min\left(Y_{ij},i=1,2,\ldots ,n\right)}$$

**4) Computation of Grey relational coefficients:** The grey relational coefficients represent the degree of similarity between the input factors and the output factor. These coefficients are calculated using the relation (7).

(7) $${GC}_{ij}=\frac{\Delta_{\min}+\lambda \;\Delta_{\max}}{\Delta_{oj}+\lambda \;\Delta_{\max}}$$

**5) Ranking the input factors:** The input factors are ranked based on their grey relational grades. A direct correlation exists between the level of grey relational grade and the strength of the linkage between the input and output factors, where a higher grade denotes a more robust relationship. Using relation (8) Grey relation grade was computed for each experiment trial. Table 6 displays the manipulated grey grade, grey coefficient value, and the S/N ratio and normalised S/N ratio.

(8) $$Grade=\frac{1}{k}\;\sum_{i=1}^{m}Y_{ij}$$

**Table 6:** S/N ratio, normalised S/N ratio, grey relational coefficients, and grey relational grade.

S/N Ratio		S/N Ratio		Sequence		Coefficient (GRC)		Grade (GRG)		Rank
(S.R)	(VAR)	(S.R)	(VAR)			(S.R)	VAR)			
−29.179	−39.965	0.62	1	0	0.38	1	0.398	0.699		3
−22.392	−42.015	0.53	0	1	0.47	0.5	0.514	0.507		9
−25.628	−43.999	0.44	0.477	0.52	0.56	0.657	0.47	0.564		7
−26.089	−33.936	0.9	0.545	0.46	0.1	0.687	0.83	0.759		2
−24.604	−38.862	0.67	0.326	0.67	0.33	0.597	0.604	0.601		6
−28.466	−53.556	0	0.895	0.11	1	0.905	0.333	0.619		5
−25.903	−31.701	1	0.517	0.48	0	0.674	1	0.837		1
−28.825	−51.373	0.1	0.948	0.05	0.9	0.95	0.357	0.654		4
−25.263	−51.373	0.24	0.423	0.58	0.76	0.634	0.395	0.515		8

### 3.2 Optimal sequence of process parameters and their levels

**Fig. 4:**
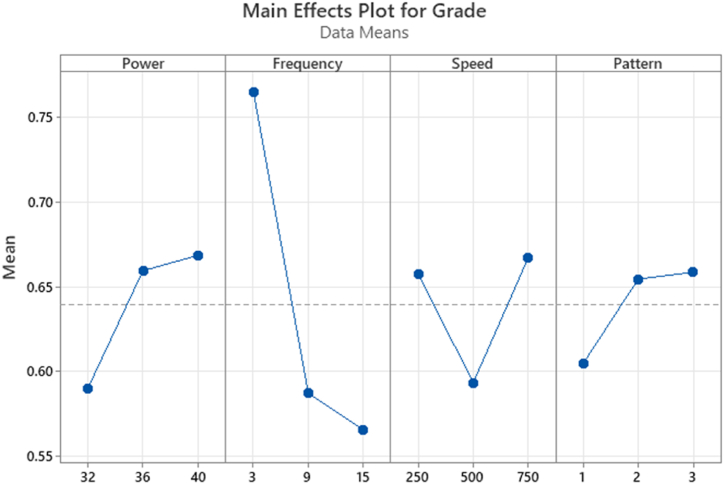
Main effect plot for GRG.

The optimal parameters for *fs*-laser micromachining to achieve minimal surface roughness and maximum volume ablation rate can be determined using GRG analysis. GRG is a grey relational grade that is obtained through the grey relational coefficient and is utilised to indicate the level of quality characteristics. Experimental runs were assigned rankings based on their GRG values. Analysis of Table 6 reveals that experimental run 7 received the premier GRG ranking, indicating satisfactory quality characteristics. Fig. 4 depicts the main effects plot for GRG, which interpretrs the optimal combination of parameters. The optimal combination of parameters for experimental run 7 includes a laser power of 40 W, pulse frequency of 3 kHz, and scanning speed of 750 mm/s. The main effect plot, which shows the relationship between the input parameters and output responses, is displayed in Fig. 8. The main effect plot demonstrates the best input parameters for femtosecond laser micromachining.

### 3.3 Analysis of variance (ANOVA) to determine the effect of parameters on the response

ANOVA analysis for surface roughness and volume ablation rate is shown in Tables 7 and 8, respectively. The ANOVA manifests the ranking significance of each parameter on the effect of responses considered. The achievement of R^2^ and R^2^ (adj) values of more than 90% reflects the suitability of the proposed model in a realistic way [36]. The ANOVA conducted for surface roughness upholds the finding that the scanning speed is the deciding parameter to control the roughness, followed by the frequency. However, the same ANOVA was conducted for the volume ablation rate, which interprets the reciprocal of surface roughness findings. Nevertheless, frequency is the most predominant parameter in controlling the volume ablation rate, followed by scanning speed. The ANOVA executed for both responses explores the insignificance of the rest of the two parameters, namely laser power and scanning pattern.

**Table 7:** ANOVA analysis for surface roughness.

Source	DF	Adj SS	Adj MS	F	P	%Contribution
Laser power	2	1.369	0.6843	0.57	0.639	3.49
Pulse frequency	2	4.935	2.4673	2.04	0.329	12.58
Scanning Speed Scanning Pattern	2 2	30.49 2.417	15.2451 1.2089	12.61	0.073	77.75 6.16
Residual Error	0	–	–			0
Total	8	39.211				100

S = 1.0995; R^2^ = 93.83; R^2^(adj) = 75.34.

**Table 8:** ANOVA analysis for volume ablation rate.

Source	DF	Adj SS	Adj MS	F	P	%Contribution
Laser power	2	6.292	3.146	6.69	0.130	1.4
Pulse frequency	2	280.752	140.376	298.47	0.003	62.7
Scanning speed Scanning pattern	2 2	159.751 0.941	79.876 0.47	169.84	0.006	35.67 0.21
Residual Error	0	–	–			
Total	8	447.736				100

S = 0.6858; R^2^ = 99.79; R^2^(adj) = 99.16.

## 4 Results and discussion

### 4.1 Effect of laser parameters on surface roughness

#### 4.1.1 Effect of laser power

**Fig. 5:**
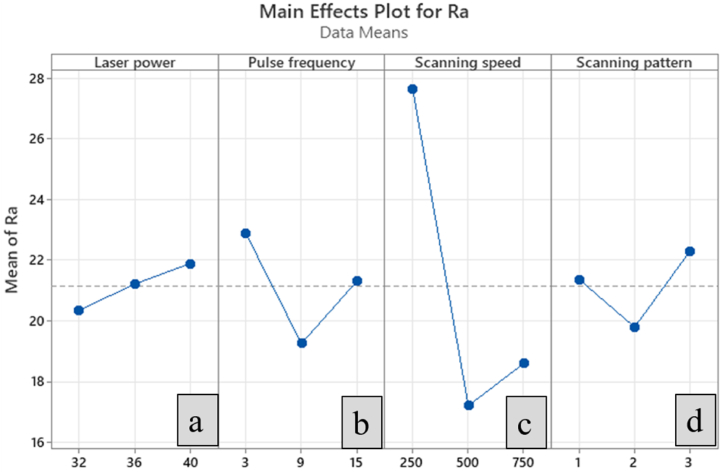
Main effect plot for surface roughness (a) Laser power (b) Pulse frequency (c) Scanning speed (d) Scanning pattern.

Fig. 5 (a) shows that the increment in surface roughness is recorded for the increased laser power. It is a known fact that laser power is linear about laser fluence (laser energy per unit area). A partial amount of excess laser energy is directed to the workpiece at high laser power. In contrast, the remaining amount of laser energy melts the surrounding material leading to the development of a Heat Affected Zone (HAZ) [37,38]. This is why an increase in surface roughness at higher laser power. With low power, adequate laser energy is generated to penetrate the workpiece entirely, ablating the material with fine quality and reducing the surface roughness.

#### 4.1.2 Effect of pulse frequency

Fig. 5 (b) shows that the higher pulse frequency and lower pulse frequency leave the stent with more surface roughness. The incremental pulse frequency limits the interval between two immediate laser pulses. The pulses strike the focused zone at a faster rate, which creates concentrated heat beyond the focused zone, leading to the formation of a Heat Affected Zone (HAZ). At a lower level of pulse frequency, the pulses strike the focused zone at a slower rate, so the pulses take a long time to vaporise the material [37,39]. During the time interval, the melted zone solidifies slightly due to the absence of laser pulses, resulting in a poor surface finish. However, at a medium level of pulse frequency, the time interval between pulses is sufficient so that the pulse can strike the focused zone intermittently with adequate thermal energy, resulting in the focused zone neither developing HAZ nor solidifying the melted zone. This balanced phenomenon of limiting the formation of HAZ and solidification of melted zone paves the way to achieve minimum surface roughness.

#### 4.1.3 Effect of scanning speed

**Fig. 6:**
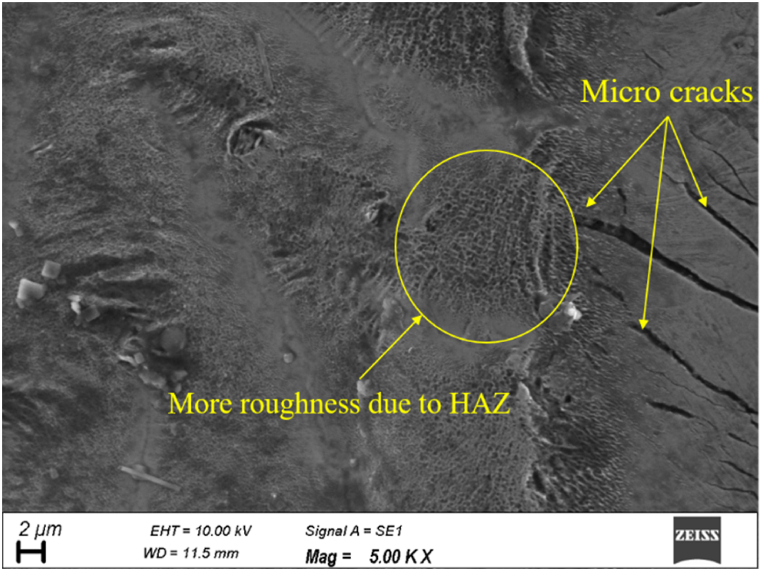
Micrograph of micromachined region at a low scanning speed.

From Fig. 5 (c), it is evident that the impact of scanning speed on the surface roughness at a lower scanning speed is worse than at a higher scanning speed. More surface roughness is recorded at low scanning speed, owing to more heat accumulated at the focused zone. The interaction time of the laser beam with the work material is more at a low scanning speed, which results in the development of accumulated heat, thereby melting the surrounding material to induce more roughness [40] and the formation of micro-cracks as depicts in Fig. 6.

Nevertheless, the interaction time between the laser beam and work material is reduced at high scanning speed, which causes the development of HAZ and, subsequently, the poorer surface. It can be seen from Fig. 5 (c) that at medium scanning speed, the surface roughness is considerably lesser than at both lower and higher scanning speeds. The medium scanning speed ensures sufficient interaction time between the laser beam and work material, which yields lesser surface roughness. The steady interaction time between the laser and work material neither develops the accumulated heat nor HAZ [38]. It can be observed from Fig. 5 (d) that the zig-zag pattern assures the lesser surface roughness followed by a concentric pattern, but the impact of linear pattern on surface roughness is worser.

### 4.2 Effect of process parameters on volume ablation rate

#### 4.2.1 Effect of laser power

**Fig. 7:**
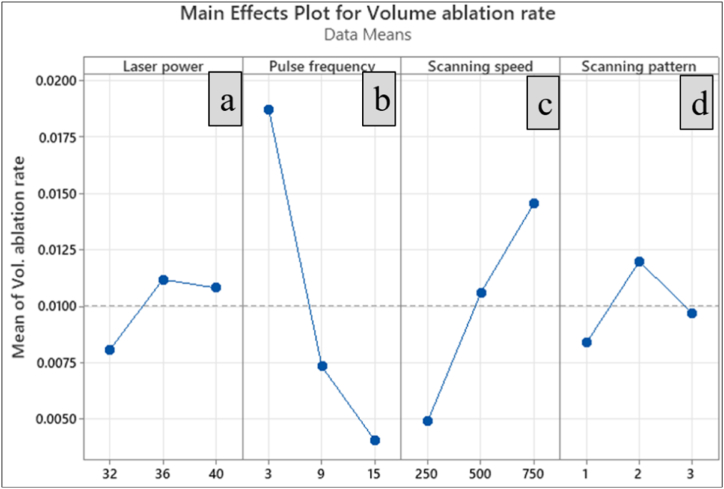
Main effect plot for volume ablation rate (a) Laser power (b) Pulse frequency (c) Scanning speed (d) Scanning pattern.

Volume ablation rate is the volume of material ablated per unit pulse during a machining process. As per ANOVA results, the laser power was found to be a significant factor that affects the volume ablation rate in laser micromachining. Fig. 7 (a) shows that the volume ablation rate is also higher at higher laser power. Since laser power is the primary energy source utilised for ablating the material, at higher levels of laser power, more energy is delivered to the workpiece [41], which ablates more material from the workpiece.

**Fig. 8:**
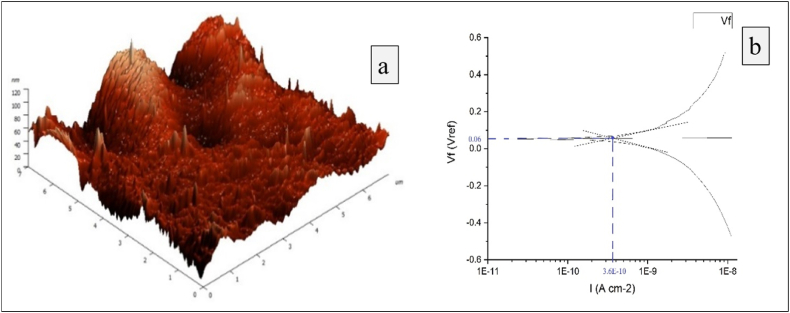
The best experimental trial (Experiment No.7) (a) Minimum surface roughness (b) Minimum corrosion.

#### 4.2.2 Effect of pulse frequency

The effect of pulse frequency is inversely proportional to the volume ablation rate, and their relationship is depicted in Fig. 7 (b). Increasing the pulse frequency drastically minimises the volume ablation rate at a constant rate owing to the thermal damage, which retards the material removal. At higher pulse frequencies, the laser pulses have enough time to induce significant heat accumulation in the material between pulses, leading to thermal damage and reduced material removal efficiency [27,41]. Also, at lower pulse frequencies, the time interval between two successive laser pulses is longer, which allows more time for heat to accumulate in the workpiece. Subsequently, the ablation rate is high owing to the more concentrated heat at the location.

#### 4.2.3 Effect of scanning speed

Fig. 7 (c) depicts that the constant increase in scanning speed linearly decreases the volume ablation rate. This is attributed to laser interaction time, where the scanning speed is inversely proportional to the laser interaction time. At minimal scanning speeds, the laser interacts with the material for a longer while, resulting in more laser pulses overlapping and creating a more effective plasma [40]. Consequently, the volume ablation rate increases. However, if the scanning speed is too low, the laser may stay in the same area for too long, leading to thermal damage and a decline in the quality of the machined feature [41]. Fig. 6 shows the micrograph manifests the development of crack caused by the thermal damage owing to the sustained laser source at low scanning speed. Conversely, the laser interacts with the material at higher scanning speeds for a shorter duration. As a result, the overlap between laser pulses decreases, making plasma formation less efficient. This drives the volume ablation rate to a decreasing trend. However, high scanning speeds can also be advantageous as they reduce thermal damage to the surrounding material and can increase the overall efficiency of the material removal process. As far as scanning pattern is concerned based on Fig. 5 (d), linear pattern helps to achieve more ablation rate followed by zig-zag pattern, but the minimal ablation rate is recorded for the concentric pattern.

### 4.3 Effect of surface roughness on corrosion rate

The experimental observation of the surface roughness after the micromachining manifests that experimental trials number 1 and 7 yield more and less surface roughness respectively. To have insight into the impact of surface roughness on corrosion behaviour, the two laser-ablated specimens belonging to experimental trials No.1 and 7 were fed into the corrosion study.

**Fig. 9:**
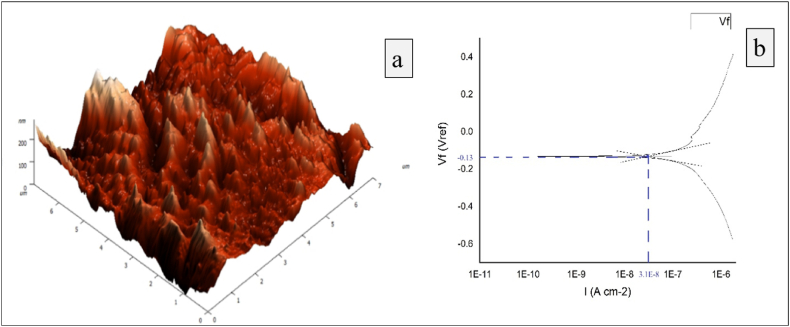
The worst experimental trial (Experiment No.1) (a) Maximum surface roughness (b) Maximum corrosion.

Based on the Tafel extrapolation method, the Tafel plot is plotted between corrosion current and corrosion potential. The upper and bottom curves represent the anodic and cathodic polarisation of the specimens correspondingly. The actual corrosion current and corrosion potential are determined with the measurement of the x and y-axis distance, respectively, at the point of intersection of two tangents drawn to both anodic and cathodic curves. Figs. 8 and 9 explore the surface roughness and corrosion behaviour from experimental trial number 7 (best) and 1 (worst) respectively. Figs. 8 (a) and Fig. 9 (a) affirm the average roughness recorded from ablated specimens are 13.171 and 28.77 nm, respectively. Similarly, Figs. 8 (b) and Fig. 9 (b) conclude the corrosion current (I_corr_) is 3.6 × 10^−10^ A/cm^2^ and 3.1 × 10^−8^ A/cm^2^ for experimental trials 1 and 7, respectively. The corrosion rates calculated using equation (2) for experimental trial numbers 1 and 7 are 9.3 × 10^−7^ mils/y and 3.49 × 10^−5^ mils/y, respectively.

Table 9 reflects the existing positive relationship between surface roughness and corrosion rate. Experimental observations reveal that the lesser surface roughness ensures a lesser corrosion rate and better corrosion resistance. The surface irregularities with larger peaks and valleys are susceptible to the formation of galvanic cells and, in turn, accelerate corrosion [42]. The earlier researcher employed two different chemical compositions of nitinol: Neostent and SeNiTi to adjudge the contribution of surface characteristics on biocompatibility and concluded that larger asperities in the surface hinder the formation of a passive layer, thereby exhibiting a poorer corrosion resistance [43]. Other researchers attempted to modify the surface of nitinol in five different textures: Non-treated, electropolished, passivated, heat-treated and air aged. They reported that the passivated and electropolished surfaces were outperformed against corrosion than the rest of all, which implied the importance of oxide layer formation and the surface smoothness [44]. However, the present study upholds the concurrence with the previous studies in minimising the corrosion behaviour but utilizes the newer methodology of mechanical machinining to minimise the surface roughness. The present findings of optimal governing parameters of *fs*-laser machining may act as guidelines to attain maximum biocompatibility for a cardiovascular stent, subsequently, the manufacturing sector will appreciate a significant cost saving with the absence of cost involved towards in-vivo tests. Apart from the stent manufacturers, industrial sectors associated with laser machining for some other bio-medical implants made out of nitinol would also benefit. Since the outcome of the study reveals exceptional and unique levels of *fs*-laser machining parameters to assure a negligible surface roughness of 10 nm, hence it can be useful to the areas where near zero surface imperfections are prominent like MEMS (Micro Electrio-Mechanical Systems) components.

**Table 9:** Comprehensive relation between surface roughness and corrosion rate.

Experiment No	Average surface roughness (nm)	Corrosion current (A/cm^2^)	Corrosion rate (mils/year)
Experiment No 1	28.77	3.1 × 10^−8^	3.49 × 10^−5^
Experiment No7	13.171	3.6 × 10^−10^	9.3 × 10^−7^

## 5 Conclusions

The present work targets the micromachining of nitinol stent using *fs*-laser machining, imitating the actual dimensions of the cardiovascular stent. The extension of the study focuses on optimising the micromachining process parameter to achieve minimum surface roughness and enhance the stent's bio-compatibility. Stringent experimental observations and mathematical analyses of experimental data draw the following significant conclusions:

1. The GRA-based mathematical model reveals that scanning speed and laser pulse frequency are significant parameters to minimise surface roughness and maximise the ablation rate.
2. ANOVA results manifest that scanning speed is the governing parameter on the two responses surface roughness and volume ablation rate, followed by pulse frequency. However, the scanning pattern was found to be a most insignificant parameter.
3. It is declared that the optimal process parameters are a laser power of 40 W, a pulse frequency of 3 kHz, and scanning speed of 750 mm/s, and a zig-zag scanning pattern.
4. The higher value of R^2^ and adjR^2^ towards unity affirms the adequacy of the suggested model.
5. The potentiodynamic polarisation technique for corrosion study at simulated body fluid medium records the minimal corrosion behaviour of the specimen whose surface roughness is minimal.
6. The experimental investigation of surface roughness and corrosion after micromachining establishes a positive relationship between surface imperfections and corrosion and also explores the improved degree of bio-compatibility of the nitinol stents.
7. As a part of the in-virto study, the improved biocompatibility has been validated through the minimal bio-corrosion achieved by a meagre surface roughness of the stent. However, further, the authentication of biocompatibility can be done either through the investigation of a thicker deposition of titanium oxide (TiO) layer to combat the nickel ions or through the study based on the in-vivo method.

## CRediT authorship contribution statement

**Venkatesh Chenrayan:** Writing – original draft, Validation, Software, Resources, Project administration, Methodology, Investigation, Funding acquisition, Data curation, Conceptualization. **Dhanabal Palanisamy:** Writing – original draft, Visualization, Validation, Software, Project administration, Funding acquisition, Data curation, Conceptualization. **Kalayarasan Mani:** Writing – original draft, Visualization, Validation. **Kiran Shahapurkar:** Writing – review & editing, Validation, Resources, Formal analysis. **Manzoore Elahi M. Soudagar:** Software, Resources, Methodology, Investigation, Conceptualization. **Yasser Fouad:** Validation, Investigation, Formal analysis, Data curation. **M.A. Kalam:** Visualization, Supervision, Resources, Project administration. **Muhammad Mahmood Ali:** Visualization, Supervision, Resources, Methodology, Funding acquisition. **Muhammad Nasir Bashir:** Visualization, Validation, Supervision, Resources.

## Declaration of competing interest

The authors declare that they have no known competing financial interests or personal relationships that could have appeared to influence the work reported in this paper.

## Nomenclature

Y_i_: Response values of ith trial
$Z_{\text{ij}}$: Normalised S/N ratio for ith^trial^
$Y_{\text{ij}}$: S/N ratio for ith^trial^
${\text{GC}}_{\text{ij}}$: Grey relation coefficient for ith^trial^
$\Delta_{\min}$: Maximum deviation sequence
$\Delta_{\max}$: Minimum deviation sequence
λ: Distinguished sequence
$\Delta_{\text{ij}}$: Deviation sequence for ith^trial^
$\gamma_{i}$: Grey relation grade for ith^trial^
